# Microwave Accelerated Transglycosylation of Rutin by Cyclodextrin Glucanotransferase from *Bacillus* sp. SK13.002

**DOI:** 10.3390/ijms12063786

**Published:** 2011-06-09

**Authors:** Tao Sun, Bo Jiang, Beilei Pan

**Affiliations:** 1 State Key Laboratory of Food Science and Technology, Jiangnan University, 1800 Lihu Avenue, Wuxi 214122, China; E-Mail: libsunt@yahoo.com; 2 Chinese Institute of Food Science and Technology, Beijing 100006, China; E-Mail: zhsuntao@hotmail.com

**Keywords:** rutin, cyclodextrin glucanotransferase, microwave-assisted reaction, transglycosylation

## Abstract

Rutin was subjected to intermolecular transglycosylation assisted with microwave irradiation using cyclodextrin glucanotransferase (CGTase) produced from *Bacillus* sp. SK13.002. Compared with the conventional enzymatic method for rutin transglycosylation (without microwave irradiation), microwave-assisted reaction (MAR) was much faster and thus more efficient. While the conventional reaction took dozens of hours to reach the highest conversion rate of rutin and yield of transglycosylated rutin, MAR of rutin transglycosylation completed within only 6 min providing almost the same conversion rate of rutin and yield of products consisting of mono-, di-, tri-, tetra-, penta-glucosylated rutins. The optimum transglycosylation conditions for microwave irradiation were 40 °C and 60 W with the reaction system consisting mainly of the mixture of 0.3 g rutin (0.49 mmol) pre-dissolved in 15 mL methanol, 1.8 g maltodextrin in 15 mL of 0.2 M sodium acetate buffer (pH 5.5) and CGTase (900 U). Results from this study indicated that MAR could be a potentially useful and economical technique for a faster and more efficient transglycosylation of rutin.

## 1. Introduction

**Figure 1:**
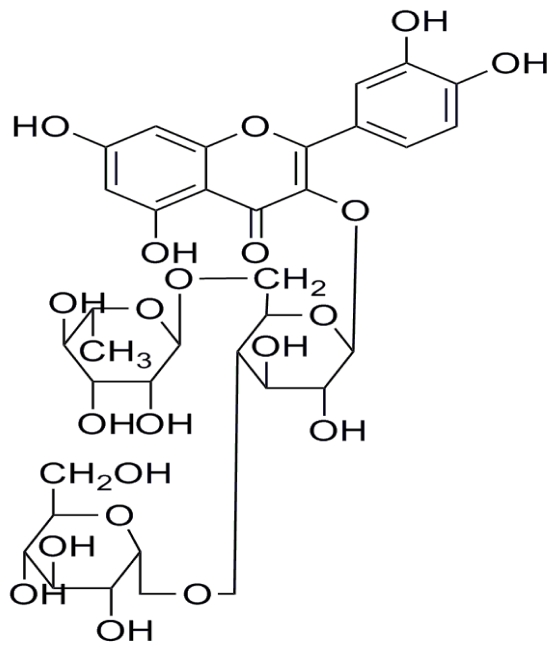
The chemical structure of glycosyl-rutin (G1-rutin).

Rutin is one kind of bioflavonoid, which is used as a nutritive element or an antioxidant in the cosmetic, food and beverage industries. However, rutin is hardly soluble in water and not very stable, which limits its practical application in industries. To improve its physicochemical properties, many methods have been investigated, including the use of CGTase to carry out enzymatic transglycosylation to obtain transglycosylated rutin with improved properties such as good water solubility [1,2]. CGTases are mainly produced by various *Bacillus* spp. and could be utilized to catalyze the enzymatic reaction which transfers glycosyl residues to compounds like rutin to form glycosyl-rutin (G1-rutin in this paper which has one glycosyl residue; its chemical structure is presented in Figure 1) and maltooligosyl- rutins with more than one glycosyl residue (G*n*-rutins, *n* is the number of glycosyl residues). These transglycosylation reactions have been carried out using conventional techniques and were reported to be time-consuming [2,3].

Furthermore, because rutin has a low solubility in water but a high solubility in methanol, the latter solvent has been used for rutin transglycosylation in order to start with higher rutin concentrations. As a result, the enzymatic transglycosylation reaction system consists of nearly half part of methanol (v/v), which might induce an inhibitory effect on CGTase activity. This would probably explain the reason why it should take dozens of hours to complete the enzymatic reaction of rutin transglycosylation if the highest conversion rate of rutin is desired.

Microwave irradiation has become an effective tool in synthetic organic chemistry, dramatically increasing reaction rates and yields [4–8]. Microwave irradiation has also been attracting more and more attention recently in enzymology for the induction and acceleration of enzymatic reactions [9–12]. However, whether or not specific microwave effects are important in biocatalysis still remains unclear [13].

Since the time-course of rutin transglycosylation was extremely long (dozens of hours), a microwave-assisted reaction (MAR) technique was investigated in this study. To the best of our knowledge, enzymatic transglycosylation of rutin under microwave irradiation conditions has not been reported so far. The present study focused on comparing the transglycosylation rate of rutin under conventional conditions with that assisted with microwave irradiation using CGTase as a biocatalyst produced from *Bacillus* sp. SK13.002 and maltodextrin as substrate to provide glycosyl residues.

## 2. Results and Discussion

### 2.1. Effect of Methanol on CGTase Used for Rutin Transglycosylation

Transglycosylation is an important process for structural modification of molecules to enhance their solubility and biological properties, which can also improve their properties and action in the body system [14–17]. In the present work, the transglycosylation of rutin was carried out using conventional and microwave-assisted techniques for obtaining transglycosylated rutin with improved water-solubility and biological properties. Previous studies on the conventional method for rutin transglycosylation (without microwave irradiation) indicated that in order to achieve about 70% conversion rate of rutin, the enzymatic reaction should take dozens of hours [1,2]. The reasons which could explain this fact are given below.

Firstly, it is well known that different enzymatic reactions would require also different reaction time periods if the highest yield of products is targeted. For the enzymatic transglycosylation of rutin, the long time-course probably depends on the nature of CGTase and the type of substrate.

**Figure 2:**
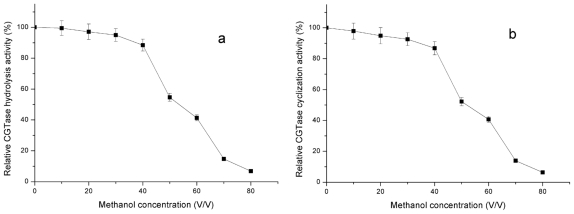
Inhibitory effect of methanol on CGTase hydrolysis activity (**a**) and on CGTase cyclization activity (**b**).

Secondly, methanol existing in the enzymatic reaction system might cause an inhibitory effect on the activity of CGTase during rutin transglycosylation. In order to test this assumption, the effect of methanol concentration on CGTase hydrolysis and cyclization activities was investigated. Results presented in Figure 2 indicate that both CGTase hydrolysis and cyclization activities are inhibited by high concentrations of methanol. In fact, when the concentration of methanol in enzymatic reaction increased, the inhibitory effect on CGTase activity increased accordingly. Moreover, at 50% (v/v) methanol, the residual CGTase hydrolysis and cyclization activities dropped to 54.6 and 52.2%, respectively. At 80% (v/v) methanol, only 6.83 and 6.31% of CGTase hydrolysis and cyclization activities remained, respectively. Thus, it might be concluded that for rutin transglycosylation, the concentration of methanol present in the enzymatic reaction system results in lower CGTase activities and consequently in a slower reaction process. Unfortunately, because rutin is hardly soluble in water, but well soluble in methanol, the latter solvent is actually essential to carry out the enzymatic reaction with a relatively high concentration of rutin. In other words, the long time-course observed for the conventional transglycosylation of rutin is almost inevitable as long as methanol exists in the reaction system.

### 2.2. Optimization of Enzymatic Conditions for Rutin Transglycosylation under Microwave Irradiation

As mentioned above, microwave irradiation might stimulate and accelerate chemical and enzymatic reactions. However, microwave irradiation did not always stimulate or accelerate chemical and enzymatic reactions [18,19]. It is unclear what role microwave irradiation plays in the enzymatic transglycosylation of rutin.

To answer this question, the effect of microwave irradiation on rutin transglycosylation was investigated. The results were positive and inspiring: Microwave irradiation could drastically accelerate the transglycosylation rate of rutin. Concretely, under specific microwave irradiation conditions, the reaction time to complete the transglycosylation of rutin decreased from dozens of hours to only several minutes.

**Figure 3:**
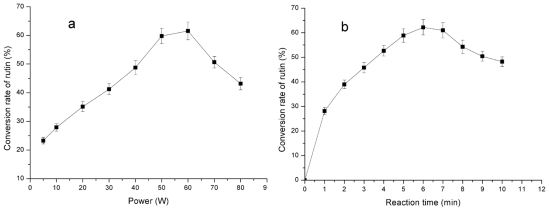
Effect of irradiation power (**a**) and time (**b**) on rutin transglycosylation in the microwave-assisted reaction (MAR) system (reaction temperature not exceeding 50 °C).

Different parameters including microwave power and reaction time were selected to standardize microwave-assisted reaction (MAR) for optimization. The effects of different microwave power levels and reaction time periods to complete the rutin transglycosylation are given in Figure 3.

The release of glycosyl residues from the substrate (maltodextrin) for transglycosylation is dependent on the availability of a particular energy level inside the microwave oven. In this study, the maximum yield of enzymatic reaction products (transglycosylated rutins) was obtained using 60 W as the irradiation power level and a 6 min reaction time.

**Figure 4:**
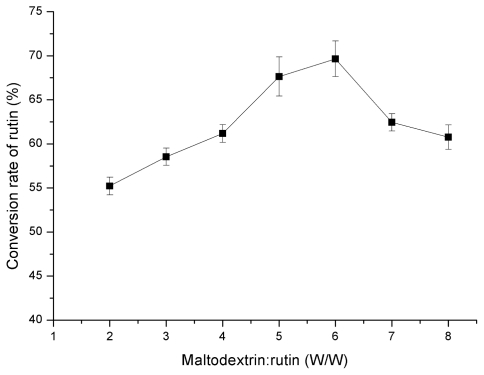
Effect of maltodextrin ratio to rutin (w/w) on rutin transglycosylation in the MAR system.

**Figure 5:**
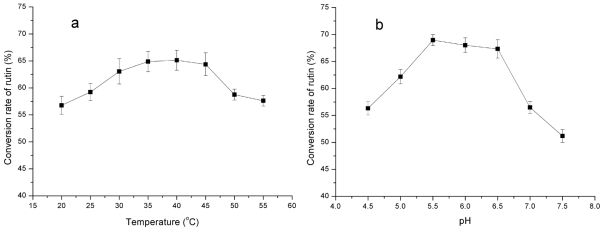
Effects of temperature (**a**) and pH (**b**) on rutin transglycosylation in the MAR system.

In order to optimize the transglycosylation of rutin under microwave irradiation conditions, the ratio of maltodextrin to rutin, as well as the reaction pH and temperature were also investigated. Results in Figure 4 showed that the optimum ratio of maltodextrin to rutin is 6:1 (w/w). In addition, the optimum pH and temperature were found to be 5.5–6.5 and 40 °C, respectively (Figure 5).

### 2.3. Comparison of Microwave-Assisted Reaction (MAR) with Conventional Enzymatic Reaction for Rutin Transglycosylation

**Figure 6:**
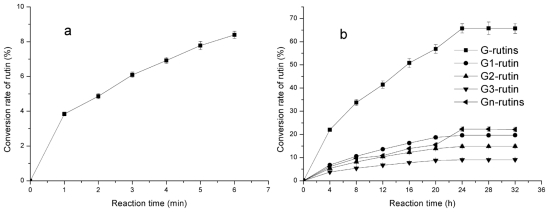
Time-courses of the conventional method for rutin transglycosylation in 0–6 min (**a**) and 0–32 h (**b**).

For the conventional transglycosylation method, reaction parameters such as pH, temperature and time were investigated in order to optimize the yield of transglycosylated rutins (G-rutins). Results revealed that the optimal pH and temperature for the conventional enzymatic reaction were 5.5 and 35 °C, respectively (data not shown). In addition, G-rutins (including G1-, G2-, G3- and Gn-rutins) increased gradually with time and reached the highest level after 24 h, but remained constant up to 32 h (Figure 6).

The optimal temperature for *Bacillus* sp. SK13.002 CGTase and other catalysis characteristics were different from other CGTases. According to Suzuki *et al*. [1], the optimal temperature of enzymatic reaction was 20 °C, while being 35 °C in this work. Furthermore, the same authors reported also that the highest conversion rate of rutin (about 70%) and production yield were obtained when the transglycosylation of rutin was carried out for 48–72 h. However, results from this study showed that 24 h would be sufficient to reach almost the same conversion rate of rutin (65.7%) and yield of transglycosylated rutins. Go *et al*. [2] stated a similar conversion rate of rutin (70.8%) but a shorter conversion time (12 h). Obviously, that shorter conversion time could be due to the lower concentration of starting rutin used in their reaction system (0.1%), whereas the starting rutin concentration in our reaction system was 10-times higher (1%). Our results indicated that the novel CGTase from *Bacillus* sp. SK13.002 has unique characteristics in terms of catalyzing capacity and optimal reaction conditions. Nevertheless, 24 h of time-course with *Bacillus* sp. SK13.002 CGTase in the conventional method for rutin transglycosylation is still too long to be economically applied. Therefore, MAR was selected as an alternative method to carry out the transglycosylation of rutin in a more competitive way.

**Figure 7:**
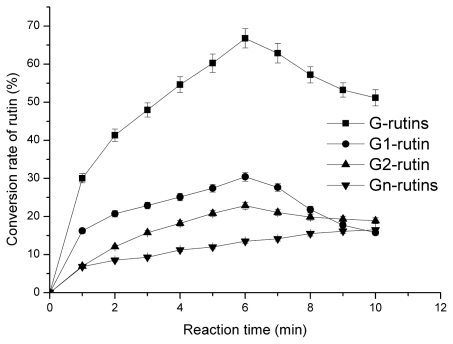
Time-course of the microwave-assisted method for rutin transglycosylation.

Compared with the conventional method for rutin transglycosylation, the microwave-assisted method dramatically accelerated the enzymatic reaction. As a result, the time required to complete the reaction decreased from dozens of hours to only several minutes (Figure 7). However, under microwave irradiation conditions, when the reaction time exceeded 6 min, the yield of G1-rutin (with one glycosyl residue) decreased markedly, while such a behavior was not observed for the conventional method. It is interesting to note that over 6 min, the rate of rutin transglycosylation was much less than that of its reverse reaction which hydrolyzes transglycosylated products, mainly G1-rutin. However, further investigations should be carried out to confirm such an assumption.

The shortening of time-course for rutin transglycosylation using MAR technique indicates a positive effect on the enzymatic reaction. Similarly, microwave irradiation has been reported to exert positive effects on different kinds of enzymes [5,12,20,21]. However, the effects of microwave irradiation on enzymatic activities were not always positive [22,23]. In many cases, microwave irradiation could also be utilized in produce and beverages in order to inactivate endogenous enzymes which might result in the deterioration of quality, and thus to prolong their shelf-life [24–26].

Although some researchers have tried to study the effects of microwave irradiation on the structure of enzymes [11,27], further studies are still required to clarify the mechanism of microwave irradiation on enzymatic reactions.

## 3. Experimental Section

### 3.1. Enzyme and Chemicals

CGTase was obtained through cultivation of *Bacillus* sp. SK13.002 which was screened and kept by ourselves. Rutin was supplied by Zelang Medical Technology Co., Ltd (Nanjing, China). Methanol and acetonitrile were obtained from Sigma (St Louis, MO, USA). Other chemicals were purchased from Sinopharm Chemical Reagent Co., Ltd (Shanghai, China).

### 3.2. Enzyme Preparation

After cultivation for 96 h, cells were removed by centrifugation at 10,000 rpm and 4 °C for 15 min. The supernatant was used as crude enzyme solution, which was concentrated using Millipore ultra-filtration system (Millipore Company, USA) with a molecular cut-off point of 10 kDa. The concentrated enzyme solution was first adjusted to pH 7.0 using solid CaCl_2_ and then precipitated with 70% saturated ammonium sulfate at 4 °C. The mixture was centrifuged at 10,000 rpm, 4 °C for 15 min and the pellets were dissolved in 20 mM Tris-HCl buffer, pH 8.0 and dialysed against the same buffer overnight at 4 °C. The solution obtained after dialysis was freeze-dried and the resulting CGTase powder was used to carry out all experiments.

### 3.3. CGTase Activity Assay

#### 3.3.1. Hydrolysis Activity of CGTase

The starch hydrolyzing activity of CGTase was assayed according to the method of Shiosaka and Bunya [28], based on the decrease of blue colour intensity of the amylose-iodine complex formed after reacting CGTase with 0.3% (w/v) soluble starch in 20 mM acetate buffer (pH 5.5) at 40 °C and measured at 660 nm. One unit of CGTase activity was defined as the amount of enzyme that catalyzed a 10% decrease of absorbance per min under the assay conditions.

#### 3.3.2. Cyclization Activity of CGTase

The cyclization activity of CGTase was tested using the phenolphthalein assay [29]. A reaction mixture containing 1 mL of 0.04 g starch in 0.1 M phosphate buffer (pH 6.0) and 0.1 mL enzyme solution was used. The mixture was incubated at 60 °C for 10 min in a water bath. The reaction was stopped by adding 3.5 mL of 0.03 M NaOH solution. After 0.5 mL of 0.02% (w/v) phenolphthalein in 0.005 M Na_2_CO_3_ was added to the reaction mixture. After 15 min, the decrease in colour intensity was measured at 550 nm. One unit of CGTase activity was defined as the amount of enzyme that forms 1 mmol β-cyclodextrin per min under the assay conditions.

### 3.4. Rutin Transglycosylation

The transglycosylation of rutin by CGTase was carried out following the method of Suzuki *et al*. [1] with a few modifications. CGTase (900 U) was incubated at 35 °C for 48 h in the dark with a mixture of 1.8 g maltodextrin pre-dissolved in 15 mL of 0.2 M sodium acetate buffer (pH 5.5), 0.3 g rutin in 15 mL of methanol and 10 mM CaCl_2_ in the final solution. The conversion rate of rutin (%) was defined as follows: Conversion rate (%) = produced G-rutins/(remaining rutin + G-rutins) × 100.

### 3.5. Microwave-Assisted Reaction

The reaction system consisted of the same components mentioned above, with the enzymatic reaction occurring in a microwave oven (2450 MHz) at 40 °C and 60 W for several minutes. The reaction was stopped by heating in boiling water for 5 min.

### 3.6. Analysis of Transglycosylated Rutin

After the transglycosylation of rutin, samples were filtered through 0.45 μm membrane and analyzed by HPLC using Agilent ZORBAX Eclipse XDB-C18 column (4.5 mm × 150 mm, 5 μm). The mobile phase contained a mixture of acetonitrile, water and formic acid (18:81.9:0.1, v/v/v) and the flow rate was 0.8 mL/min. UV detector was used at 254 nm, with the column temperature being 30 °C.

### 3.7. Statistical Analysis

All experiments were performed in triplicate and results are expressed as mean ± standard deviation.

## 4. Conclusions

In the enzymatic transglycosylation of rutin, methanol used as solvent causes an inhibition of CGTase activities, which might result in dozens of hours of reaction time to achieve the highest conversion rate of rutin and yield of transglycosylated products, which are mono-, di-, tri-, tetra-, penta-glucosylated rutins when maltodextrin is used as the donor of glycosyl residues.

Microwave irradiation accelerated dramatically the enzymatic reaction rate during rutin transglycosylation. Using 60 W at 40 °C in microwave-assisted reaction (MAR) system, the transglycosylation of rutin completed within only 6 min. Therefore, MAR could be a potentially useful and economical technique for a faster and more efficient transglycosylation of rutin.
